# Clinical efficacy of DSA-based features in predicting outcomes of acupuncture intervention on upper limb dysfunction following ischemic stroke

**DOI:** 10.1186/s13020-024-01026-5

**Published:** 2024-11-09

**Authors:** Yuqi Tang, Sixian Hu, Yipeng Xu, Linjia Wang, Yu Fang, Pei Yu, Yaning Liu, Jiangwei Shi, Junwen Guan, Ling Zhao

**Affiliations:** 1 School of Acu-Mox and Tuina, Chengdu University of Traditional Chinese Medicine, Chengdu, 610075 China; 2 National Clinical Research Center for Chinese Medicine Acupuncture and Moxibustion, First Teaching Hospital of Tianjin University of Traditional Chinese Medicine, Tianjin, 300074 China; 3 Department of Radiology, West China Hospital, Sichuan University, Chengdu, 61004 Sichuan China; 4 Chongqing Traditional Chinese Medicine Hospital, Chongqing, 400021 China; 5 Department of Neurology, Hospital of Chengdu University of Traditional Chinese Medicine, Chengdu, 610072 China; 6 Neurosurgery Department, West China Hospital, Sichuan University, Chengdu, 61004 Sichuan China

**Keywords:** Stroke, Machine learning, DSA, Object detection, Predictive model, Upper limb dysfunction

## Abstract

**Background and objectives:**

This study aimed to employ machine learning techniques to predict the clinical efficacy of acupuncture as an intervention for patients with upper limb motor dysfunction following ischemic stroke, as well as to assess its potential utility in clinical practice.

**Methods:**

Medical records and digital subtraction angiography (DSA) imaging data were collected from 735 ischemic stroke patients with upper limb motor dysfunction who were treated with standardized acupuncture at two hospitals. Following the initial screening, 314 patient datasets that met the inclusion criteria were selected. We applied three deep-learning algorithms (YOLOX, FasterRCNN, and TOOD) to develop the object detection model. Object detection results pertaining to the cerebral vessels were integrated into a clinical efficacy prediction model (random forest). This model aimed to classify patient responses to acupuncture treatment. Finally, the accuracies and discriminative capabilities of the prediction models were assessed.

**Results:**

The object detection model achieved an optimal recognition rate, The mean average precisions of YOLOX, TOOD, and FasterRCNN were 0.61, 0.7, and 0.68, respectively. The prediction accuracy of the clinical efficacy model reached 93.6%, with all three-treatment response classification area under the curves (AUCs) exceeding 0.95. Feature extraction using the prediction model highlighted the significant influence of various cerebral vascular stenosis sites within the internal carotid artery (ICA) on prediction outcomes. Specifically, the initial and C1 segments of the ICA had the highest predictive weights among all stenosis sites. Additionally, stenosis of the middle cerebral, anterior cerebral, and posterior cerebral arteries exerted a notable influence on the predictions. In contrast, the stenosis sites within the vertebral artery exhibited minimal impact on the model's predictive abilities.

**Conclusions:**

Results underscore the substantial predictive influence of each cerebral vascular stenosis site within the ICA, with the initial and C1 segments being pivotal predictors.

## Introduction

Stroke remains the leading cause of mortality and severe disability worldwide, with ischemic stroke (IS) being the most common form. Due to aging of the global population, the impact of stroke is progressively increasing [1]. Over the past three decades, advancements in stroke prevention, treatment, and rehabilitation have led to a decline in its incidence and mortality rates; however, the overall global burden of stroke continues to rise [2]. In China alone, the annual incidence of stroke has risen notably, with approximately 1.5–2 million new cases reported annually in the past decade [3]. Neurological dysfunction is a common clinical manifestation of patients with IS, with studies indicating that nearly 37% of patients experience acute upper limb paralysis post-stroke, with 5% continuing to suffer from issues such as reduced upper limb control and fine motor function of the hand three months later. Approximately 60% of stroke survivors exhibit varying degrees of upper limb motor dysfunction six months after onset [4]. This symptom significantly impacts patients' activity levels and quality of life, presenting ongoing challenges in rehabilitation [2].

Under conventional treatments, the recovery of upper limb motor dysfunction following stroke remains suboptimal. both primary and secondary injuries can hinder the recovery of upper limb motor function during various stages of rehabilitation [5]. Research has indicated that acupuncture therapy effectively treats hemiplegia post-stroke, offering high safety, minimal side effects, low economic burden, and high patient acceptance. Acupuncture has further been widely integrated into stroke motor dysfunction rehabilitation [6, 7]. Studies have highlighted the efficacy of acupuncture in stimulating neural plasticity, thereby facilitating brain motor function recovery [8]. The restoration of motor function in IS patients hinges on reestablishing cerebral blood circulation, including collateral circulation, and reperfusing the ischemic penumbra. However, the evidence supporting the role of acupuncture in enhancing cerebral blood circulation recovery remains insufficient to achieve a conclusive result. Digital subtraction angiography (DSA), the gold standard in cerebrovascular disease diagnosis, plays a pivotal role in the diagnosis of IS [9]. Unlike computed tomography (CT) and magnetic resonance imaging (MRI), which are mainly used to identify the location and extent of lesions, DSA can clearly show the stenosis and occlusion of cerebral vascular network, microcirculation, and collateral circulation [10]. Further, DSA can allow the comprehensive visualization of cerebral blood supply. As such, DSA can not only achieve rapid and accurate diagnosis, but can also help doctors to accurately judge disease progression, develop treatment plans, and analyze prognosis. Using DSA, changes in brain function can be comprehensively visualized from various perspectives such as the vascular network, collateral circulation establishment, and microcirculation. This approach provides insights into cerebral vessel structural alterations and reveals the effects of acupuncture on post-stroke motor dysfunction.

In recent years, machine learning technologies have progressed rapidly owing to the availability of complex medical big data. As such, these technologies have been increasingly employed in the diagnosis, classification, image analysis, and risk prediction of various diseases [11]. Medical image-assisted diagnosis represents a crucial application domain, particularly in intelligent image recognition, personalized diagnosis support, human–computer interaction-assisted diagnosis, and precise treatment decision-making [12]. Machine learning has further achieved considerable maturity in stroke neuroimaging analysis, capable of extracting pertinent imageomics features from diverse neuroimaging modalities, such as CT and MRI. However, only a few studies have investigated the specific pathological vascular features relevant to IS patients [13]. Therefore, in the present study, we employed machine learning techniques, leveraging object detection technology to extract valuable feature data from DSA imaging. These data were integrated with the diagnostic and treatment records to develop an efficacy prediction model. This model aimed to guide prognostic evaluation and predict treatment responses to acupuncture therapy in patients with upper limb motor dysfunction following IS.

## Methods

### Sample data

#### Standard protocol approvals and registrations

Sample data were obtained from the Hospital of Chengdu University of Traditional Chinese Medicine and the Fifth People's Hospital of Chengdu. The research protocol was reviewed and approved by the Medical Ethics Committee of the Hospital of Chengdu University of Traditional Chinese Medicine (Ethics Approval No. 2023KL-023).

#### Inclusion and exclusion criterias

Patients were diagnosed according to the diagnostic criteria in the International Classification of Diseases (ICD-11) and the Chinese guidelines for the diagnosis and treatment of acute ischemic stroke, 2018 [14]. The inclusion criteria were as follows: (1) Clear diagnosis of ischemic stroke through imaging examination (MRI or CT), with a course of 1–14 days; (2) First instance of unilateral hemispheric ischemic stroke; (3) Age range from 18 to 80 years old, regardless of gender; (4) No head surgery or thrombolysis and thrombectomy treatment following disease onset; (5) Clear upper limb dysfunction (Brunnstrom stage II-V). (6) Receipt of standardized acupuncture treatment during the acute phase, with a treatment period exceeding 14 days (7). Performance of DSA within 7 days after hospital admission. The exclusion criteria were as follows: (1) significant amount of missing medical record data or image data resulting in the participants not meeting the research requirements and (2) presence of other serious organic diseases that may cause limb dysfunction, such as multiple sclerosis, traumatic brain, or spinal cord injury.

The standard acupuncture treatment was based on secondary stroke prevention [15] and basic nursing care and adopts the treatment of *Xingnao Kaiqiao* acupuncture [16]. Secondary stroke prevention treatment included respiratory support, anti-platelet aggregation, improving brain blood circulation, neuroprotective therapy, risk factor control, prevention of infection, and treatment of complications [17]. The main acupoints used for *Xingnao Kaiqiao* acupuncture were PC6 (Neiguan), DU26 (Shuigou), SP6 (Sanyinjiao), DU20 (Baihui), GB20 (Fengchi), LU5 (Chize), HT1 (Jiquan), and LI4 (Hegu). According to the actual situation of patients with upper limb dysfunction, it is possible to add or subtract acupoints; however, such changes must conform to the syndrome differentiation of the meridians in the upper limb dysfunction region. As such, it is necessary to arrange trained professional acupuncture doctors to screen the data in the subsequent data processing stage.

#### Clinical evaluation and grouping

This study utilized the Fugl-Meyer Assessment of the Upper Extremity (FMA-UE) as an efficacy evaluation indicator. The FMA-UE is widely recommended and recognized as a reliable scale for assessing upper limb motor dysfunction following stroke, providing a comprehensive evaluation of motor function [18]. Patient motor dysfunction was evaluated using the FMA-UE scores obtained at admission and discharge. Data were extracted from the evaluations conducted at the time of admission and discharge. Patients were categorized based on the change in the FMA scores between admission and discharge. Following the criteria from relevant literature and clinical expertise [19–21], we defined the minimal clinically important difference (MCID) as four points, with a double MCID serving as the criterion for a high response to acupuncture intervention. Patients with FMA-UE ≥ 8 was enrolled in the high response group, patients with 4 ≤ FMA-UE < 8 in the low response group, and patients with FMA-UE < 4 in the non-response group.

### DSA image data preprocessing

We classified and coded the anatomical sites in the DSA images strictly following DSA cerebrovascular segmentation. The main contents of the classification were as follows: (1) Aortic arch classification; (2) The C1-C7 segment of the left/right Internal Carotid Artery (ICA), the beginning of the ICA; (3) The M1-M4 segment of left/right Middle Cerebral Artery (MCA), the beginning of the MCA; (4) The A1-A5 segment of left/right Anterior Cerebral Artery (ACA), the beginning of the ACA; (5) The V1-V5 segment of the left/right Vertebral Artery (VA), the beginning of the VA; (6) The P1-P4 segment of the left/right Posterior Cerebral Artery (PCA); (7) Left/right Common Carotid Artery (CCA); (8) Left/right Subclavian Artery (SCA); (9) Base artery; (10) The circulation of the MCA; (11) Circulation of the ACA. Initially, the clearest DSA images meeting the specified criteria were selected based on the aforementioned classification. Subsequently, the image annotation software “labellmg” was employed to annotate cerebral blood vessel stenosis sites across all DSA images. The stenosis sites were graded based on the stenosis ratio of the patient. According to relevant literature [22, 23], ≤ 30% was classified as mild, 30 < stenosis ratio < 69 as intermediate, 70 ≤ stenosis ratio < 99 as severe, and stenosis ratio = 100% as occlusive.

### Establishment of sample data

Sample data were gathered from the medical records and diagnostic reports of inpatients in the Neurology Departments of the two hospitals. A total of 735 patients who experienced ischemic stroke between 2020 and 2024 were included in the study. The dataset comprised three main components: demographic information (sex and age), DSA image data, and efficacy evaluation indices (FMA-UE).

**Fig. 1:**
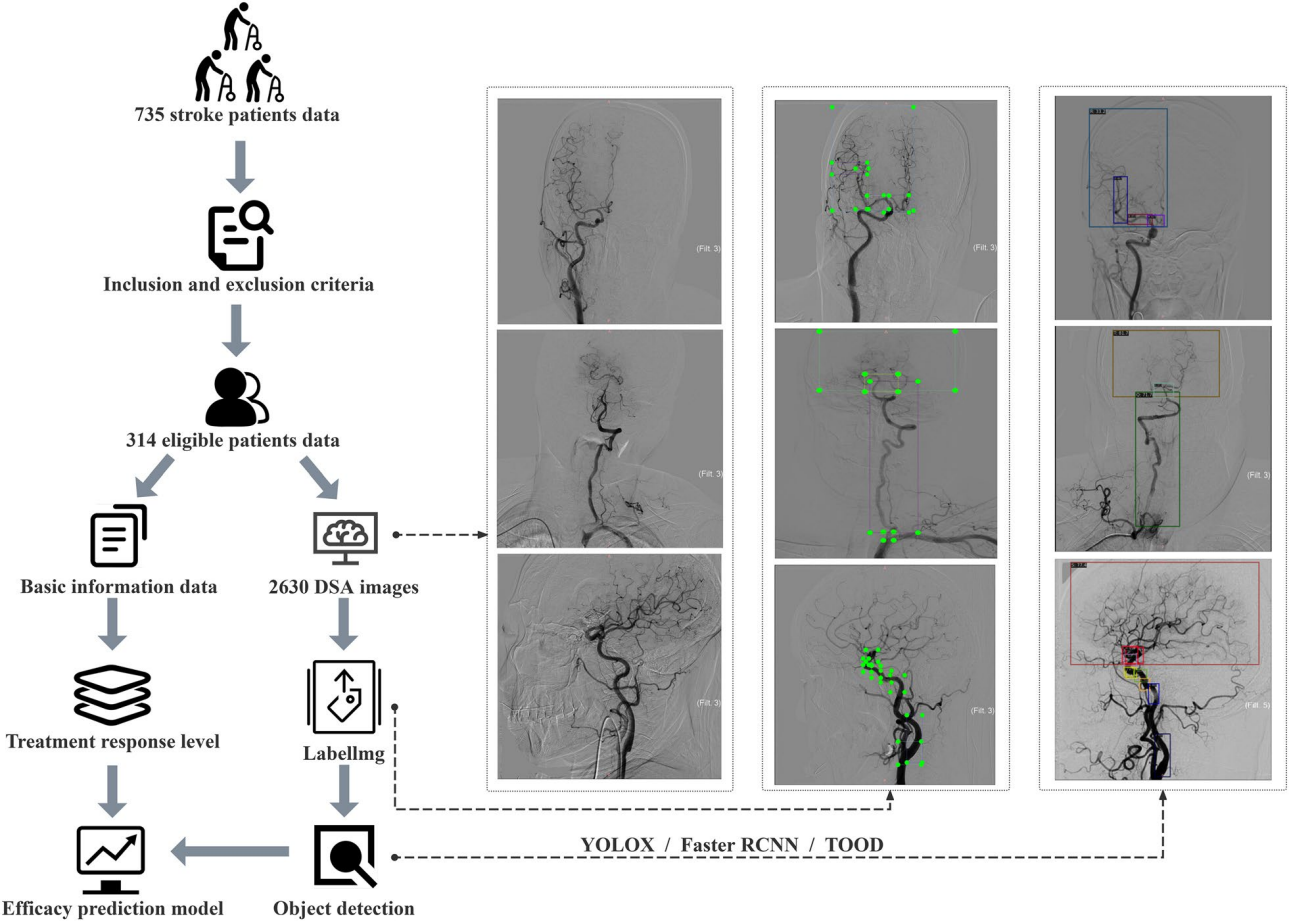
Research flow chart. The green annotation in the figure indicates the location of cerebral vascular stenosis in the DSA image, as annotated by the image annotation software "LabelImg" The other colored annotations represent the locations of cerebral vascular stenosis identified through object detection technology (YOLOX, Faster RCNN, and TOOD)

Three specialists screened data from 735 stroke patients in strict adherence to the predefined inclusion and exclusion criteria. Ultimately, the datasets of 314 stroke patients met the criteria and were processed. According to relevant literature, achieving effective classification prediction results for brain imaging data typically necessitates more than 100 images per category when the number of categories exceeds 10. In this study, a total of 2,630 digital subtraction angiography (DSA) images were collected from 314 patients, with each image containing multiple cerebral vascular classifications. This dataset provides a robust foundation for subsequent data analysis [24, 25]. Primary data, including patient information and DSA images, were anonymized to protect patient privacy. During screening, workload distribution was shared equally between the two radiologists, followed by cross-validation upon completion to mitigate subjective biases and errors. Any discrepancies encountered during cross-validation were resolved by a senior physician. After correction, Tukey's multiple comparison test was utilized to statistically assess differences in treatment outcomes before and after intervention for patients. And the dataset was compiled and imported into Python for further analysis. Initially, this study utilized object detection models (YOLOX, Faster RCNN, and TOOD) to identify constricted areas in blood vessels from DSA images and categorize stenosis severity. Subsequently, both the basic information data and object detection results were integrated into the final efficacy prediction model. This model aimed to classify patients’ responses to acupuncture treatment. The research design is presented in Fig. 1.

### Object detection and deep learning model

Object detection on the DSA image data was conducted using the YOLOX, FasterRCNN, and TOOD models. The research dataset includes two types of files: original image data and annotation files. The original image data consisted of DSA images in PNG format that required labeling. The annotation files specify the precise locations of objects within the images, with manual annotations saved in the XML format. Two neurologists were responsible for the image acquisition and annotation using the Labellmg tool. Data augmentation was performed, and the dataset was randomly divided into the training, test, and validation sets at an 8:1:1 ratio. The accuracies, recalls, and average accuracies of the three models were subsequently evaluated. The optimal model identified through these evaluations was used as an imaging feature in the efficacy prediction model to detect cerebral vascular stenosis.

**Fig. 2:**
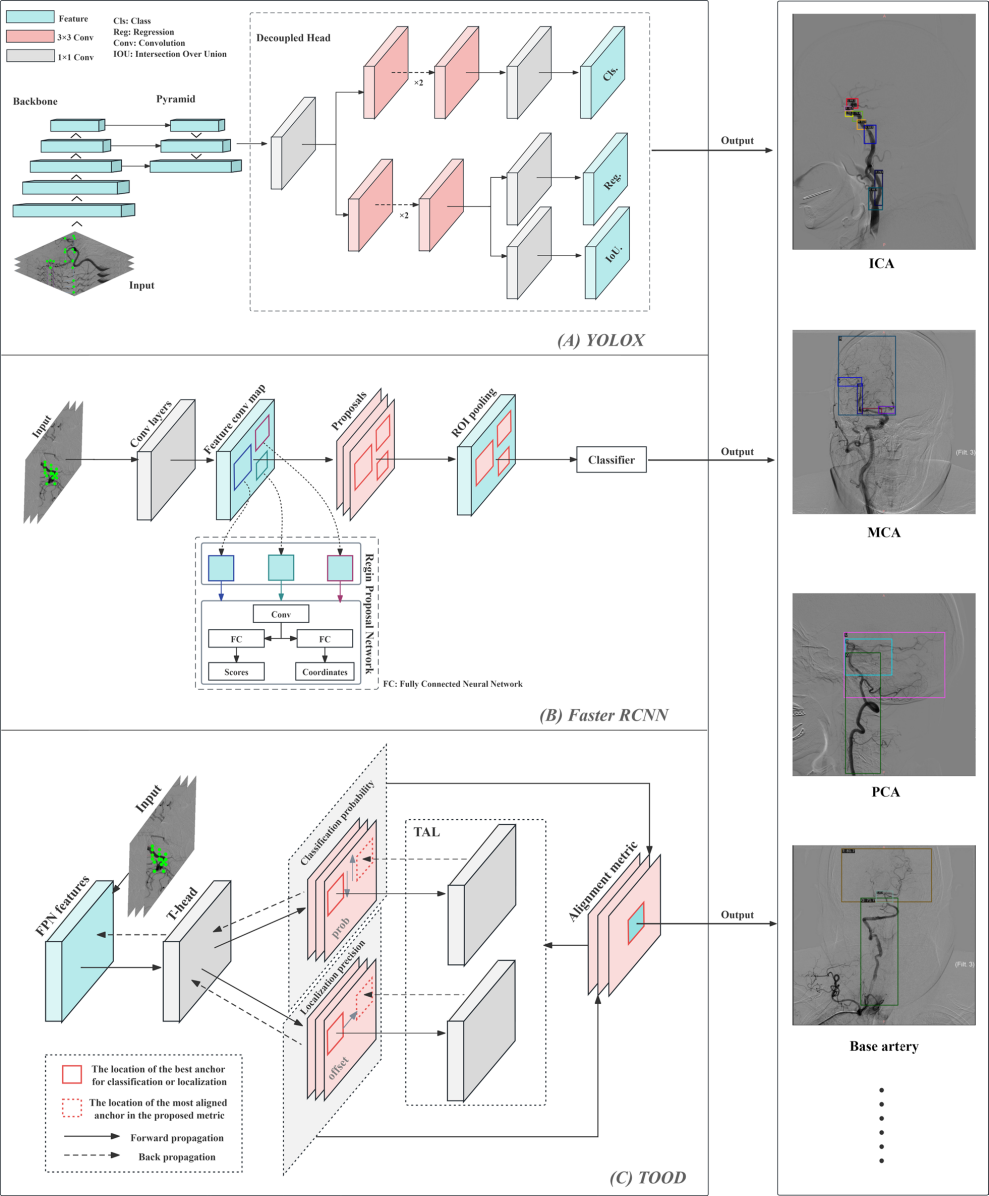
Detailed architecture of object detection models

YOLOX is an object detection model known for its excellent accuracy and speed, utilizing convolutional neural networks to detect and classify objects in images and videos [26]. YOLOX significantly improves upon previous YOLO models by introducing an anchor-free design and a decoupled head structure, in addition to enhanced data augmentation methods. This one-stage detector comprises a backbone, a neck, and a decoupled head. YOLOX extracts a feature map from an input image by using a backbone. The neck, which connects the backbone and head, includes spatial pyramid pooling (SPP) and a feature pyramid network (FPN) [27, 28]. The model architecture used in this study is presented in Fig. 2A.

Faster R-CNN employs a region proposal network (RPN) instead of a selective search, which reduces detection time and enhances target detection. The architecture of the Faster R-CNN is shown in Fig. 2B. The detection process involved the following steps: The CNN extracts feature from the image, the RPN generates candidate regions of various sizes at each location on the feature map, and the region proposals are screened. Using region of interest pooling, the CNN feature map was integrated with the region proposals generated by the RPN, followed by the classification and localization of objects through regression [29].

The Task-Aligned One-Stage Object Detection (TOOD) strategy explicitly aligns detection and classification tasks in a learning-based manner [30]. The two critical components of this architecture are the Task-aligned Head (T-head) and Task Alignment Learning (TAL). T-Head offers a better balance between learning task-interactive and task-specific features, and greater flexibility in learning alignment via a task-aligned predictor. TAL explicitly unifies the optimal anchors for the two tasks during training, using a designed sample assignment scheme. The basic architecture of TOOD is shown in Fig. 2C.

### Construction and evaluation of efficacy prediction model

The random forest (RF) framework was applied to construct a prediction model using basic demographic (age and sex) and DSA imaging (stenosis location and ratio) data. The prediction endpoint was the response level of the patients after treatment, as measured using the FMA-UE. The dataset was randomly divided into the training, testing, and validation sets at a ratio of 8:1:1, and threefold cross-validation was applied. The AUC value was further applied to assess the discriminative ability of the prediction model, whereas the Micro-F1 score was used to evaluate the accuracy of the model.

RF is an ensemble learning method comprising multiple decision trees which can extend classification and regression trees by modeling the response variable through recursive partitioning, where the trees collaborate to predict the final results. There are three mainstream decision tree algorithms: ID3, C4.5, and CART. In the present study, the widely used CART algorithm was used to construct the RF model. The primary formula used in the algorithm was as follows:

Suppose there is a training dataset D with k classes in total. The Gini index of set D is expressed as follows:

$$Gini(D) = \sum\limits_{k} {\frac{{|C_{k} |}}{D}} (1 - \frac{{|C_{k} |}}{D}) = 1 - \sum\limits_{k} {(\frac{{|C_{k} |}}{|D|})^{2} }$$

## Results

### Basic information

**Fig. 3:**
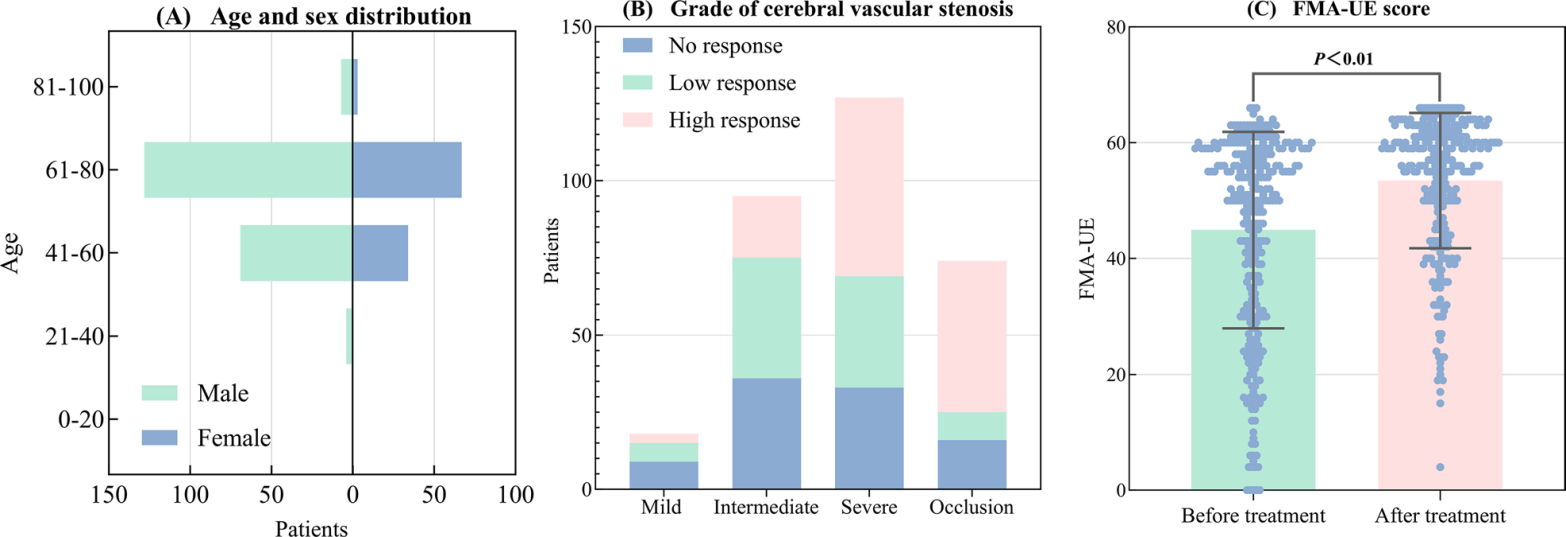
Demographic data

Basic information, including age, sex, degree of cerebral vascular stenosis, and response to acupuncture treatment, was collected. As shown in Fig. 3A, most of the patients in this study were male, with ages ranging from 40 to 80 years. As shown in Fig. 3B, cerebral vascular stenosis in these patients was predominantly intermediate to severe. Furthermore, as the degree of cerebrovascular stenosis increased, both the level and proportion of patient responses to acupuncture treatment increased. As shown in Fig. 3C, there is a significant difference in FMA-UE before and after acupuncture treatment for patients.

### Object detection results of cerebral vascular stenosis sites

**Fig. 4:**
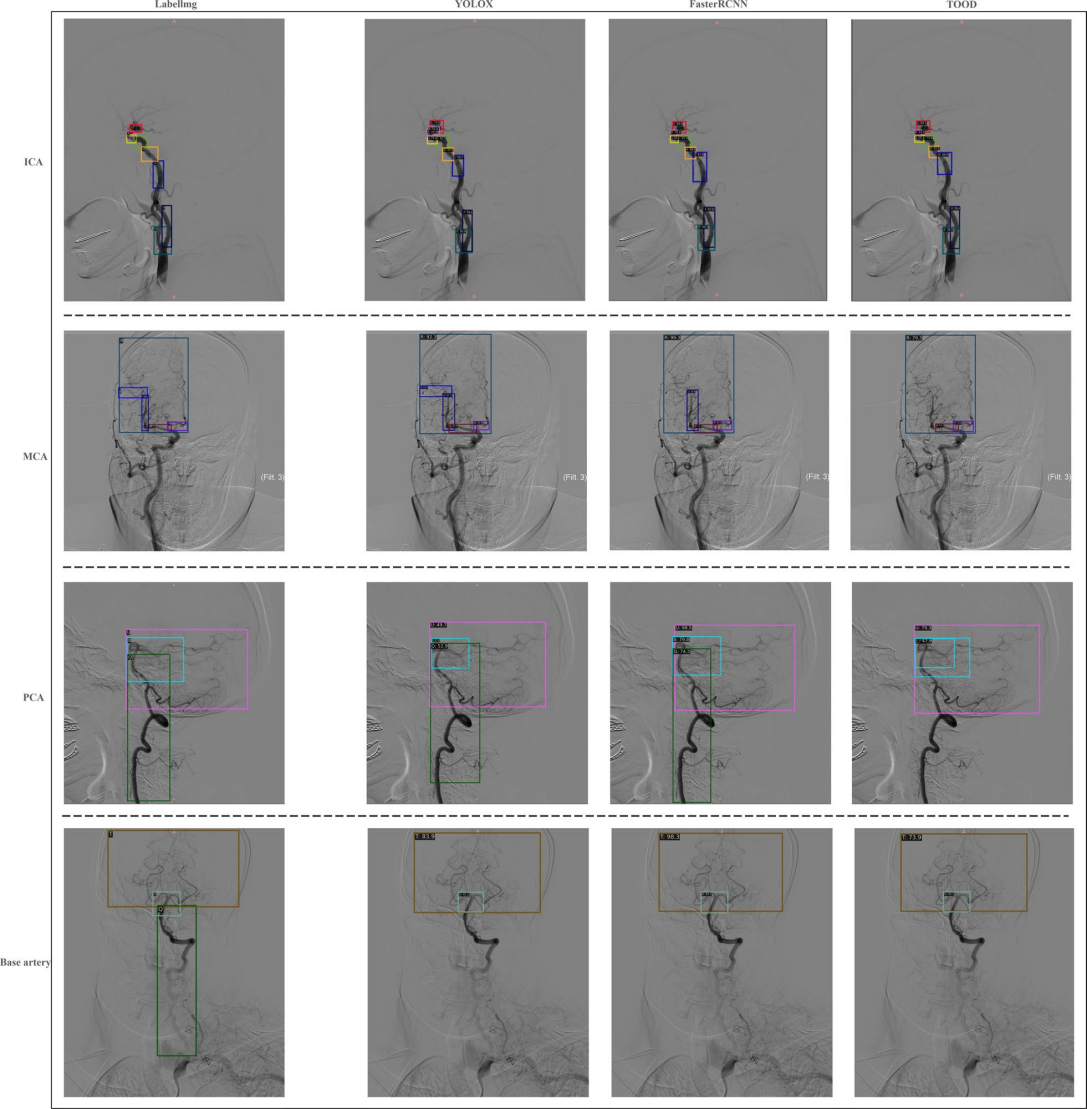
Sample results of the object detection models

In this study, three object detection models were constructed to identify stenosis sites in cerebral blood vessels from DSA images of patients. Random samples were further obtained from the identification of each blood vessel type. The sampling results for the key cerebral blood vessels (ICA, MCA, PCA, basilar artery) are shown in Fig. 4. The results of this analysis indicated that all three object detection models effectively identified the different stenosis sites with high accuracy. However, each model exhibited varying degrees of object loss, likely due to the large number of segmented cerebral blood vessel sites and the uneven distribution of the sample data. To ensure the comprehensive characterization of stenosis sites for the efficacy prediction model, we selected the highest confidence recognition results from the three models for each type of stenosis site for use in further research. In addition, in the target detection results, we identified cases of repeated detection of the same label target in one image, as shown in Fig. 4 for the recognition results of TOOD on PCA. Because the shape of each patient's cerebral blood vessels was not exactly the same, and even if some patients' cerebral blood vessels were excessively curved, there may be high similarity labels in the same image. In subsequent research, it was possible to reduce the occurrence of repeated detection using higher-precision labels or by determining a better labeling scheme during the label creation process and creating a golden dataset to reduce the occurrence of repeated detection.

#### Comparison and evaluation of object detection models

**Fig. 5:**
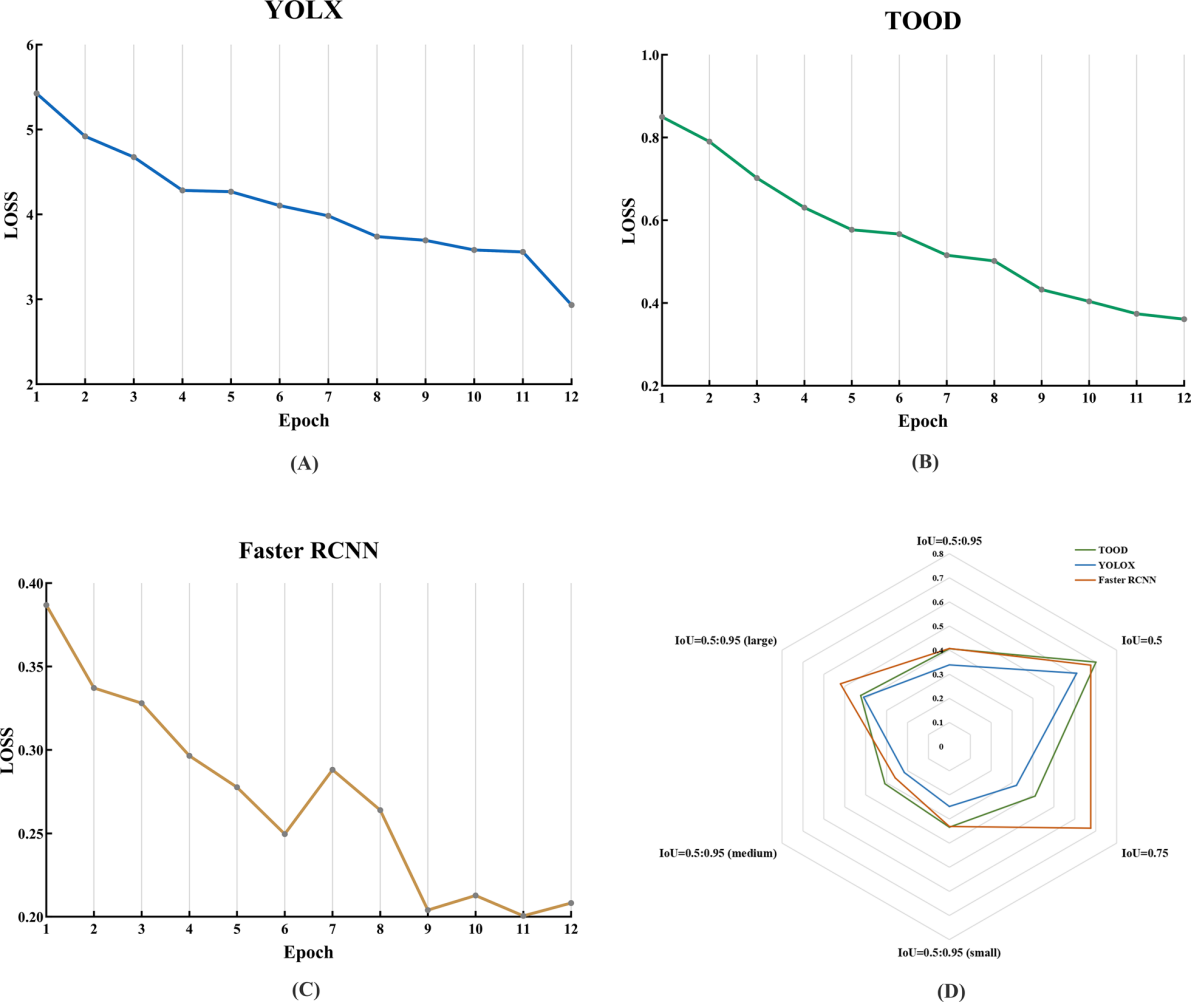
Comparison of object detection models

We plotted the loss function curves of the three object detection models over the first 12 iterations (Fig. 5A–C). As shown in the training comparison graph, the loss function curves converged, and the loss values decreased and stabilized with an increasing number of iterations, indicating a minimal difference between the model predictions and true labels. Figure 5D shows the comparison of the mean Average Precision (mAP) of the three object detection models at different IoU thresholds (0.5, 0.75, and 0.95). Overall, Faster RCNN demonstrated relatively balanced performance, achieving an mAP of 0.68 at IoU = 0.75. Additionally, at IoU = 0.5, TOOD and YOLOX achieved mAPs of 0.7 and 0.61, respectively. The poor performance of YOLOX may have resulted from its insufficient ability to detect small targets and inaccurate bounding box positioning. Furthermore, a large number of labels on the same image may affect the recognition performance of each model.

### Clinical efficacy prediction model

**Fig. 6:**
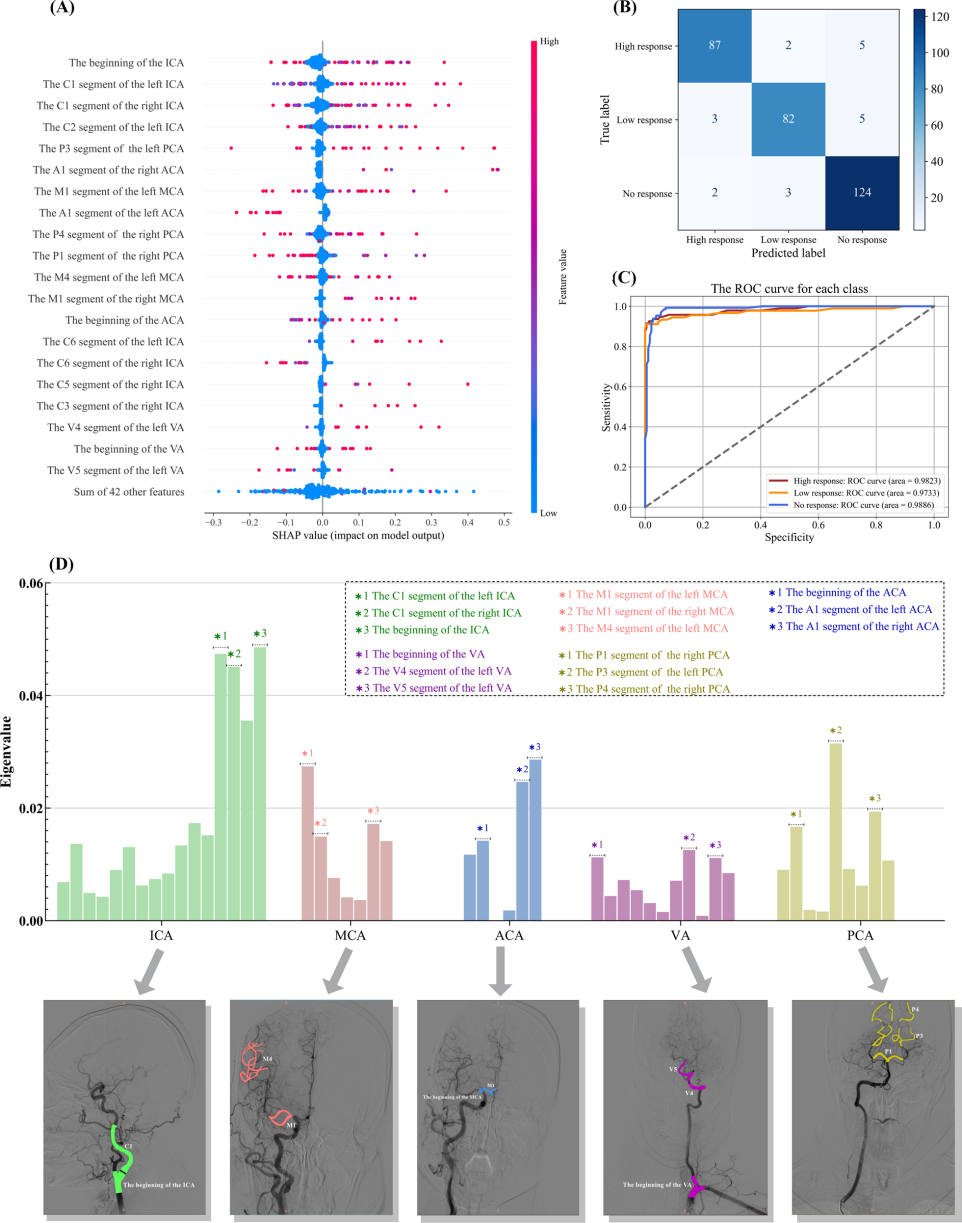
Results and evaluation of clinical efficacy prediction model. Panel **A** presents a beeswarm plot depicting the features of cerebrovascular stenosis sites. Panel **B** displays the confusion matrix of the predicted results. Panel **C** shows the ROC curve of the predictive model, and Panel **D** illustrates the feature weight ranking map for cerebrovascular stenosis sites

The input features for the clinical efficacy prediction model included patient age, sex, location of cerebrovascular stenosis (as determined by the object detection), cerebrovascular stenosis ratio, and cerebrovascular stenosis level. The predictive endpoint was the patient's response to acupuncture treatment, which was categorized as high, low, or no response. The confusion matrix for the prediction results is shown in Fig. 6B. The prediction accuracy was 93.6%.

#### Feature extraction of cerebral vascular stenosis location

The features of each cerebrovascular stenosis site were extracted and ranked according to their weights in the efficacy prediction model, as shown in Fig. 6D. The top three stenosis sites in each of the five cerebral vascular classifications (ICA, MCA, ACA, VA, and PCA) were analyzed. The stenosis sites in the ICA exhibited the greatest influence on the model's prediction results, with the beginning of the ICA and C1 segment of the ICA having the highest weights. The top three stenotic sites all had balanced weights in the MCA, ACA, and PCA classifications, indicating a significant influence on the prediction results. Conversely, the low weight of the stenosis sites in the VA had minimal influence on the model predictions.

In summary, the ICA is a key indicator for assessing the efficacy of acupuncture interventions for limb motor dysfunction post-IS. The MCA (particularly the M1 and M4 segments), ACA (the beginning and A1 segments), and PCA (the P1, P3, and P4 segments) also significantly affected efficacy. Although the VA (beginning, V4, and V5 segments) had some influence, this was less pronounced. Further, although age and sex were important factors in the efficacy prediction model, a horizontal comparison was not conducted because of the significant differences in the data distribution between these demographic features and the cerebral vascular stenosis site features.

#### Evaluation of the clinical efficacy prediction model

Figure 6C presents the receiver operating characteristic (ROC) curve of the model. The area under the curve (AUC) values for the three classification predictions were all above 0.95, indicating strong discriminative ability. In addition, the F1-micro score of the model was 0.94, demonstrating its high accuracy and reliability of the prediction model.

We further introduced Shapley additive explanations (SHAP) to elucidate the prediction model. SHAP, which is grounded in the optimal Shapley value from game theory, explains individual predictions. We focused on the top 20 features and constructed a beeswarm plot (Fig. 6A). Each point in the plot represents the shape of a feature for a particular instance. The y-axis denotes the feature and the x-axis represents the Shapley value. The results indicated that the higher the importance of a feature, the higher its Shapley value and distribution density. Notably, for the top five features (ICA), there was a strong positive correlation between the feature value and the Shapley value.

## Discussions

Individualized acupuncture treatment has been a major focus of research in recent years. By considering the basic pathological characteristics of the disease and the specific pathological characteristics of different patients, selecting an optimal treatment strategy can improve efficacy, reduce the waste of medical resources, and alleviate patients' economic burdens. This study hypothesized that IS patients with different cerebral vascular stenosis sites may respond differently to acupuncture intervention during the recovery of upper limb motor function. Based on this hypothesis, we employed three deep learning object detection techniques (YOLOX, Faster R-CNN, and TOOD) to locate cerebral vascular stenosis sites and constructed a clinical efficacy prediction model using a RF algorithm with these sites as features. The model successfully predicted the response of patients with post-stroke upper limb motor dysfunction to acupuncture intervention and analyzed the impact of different stenosis sites on the efficacy of the intervention using feature extraction values.

The results indicated that all five types of cerebrovascular disease influenced the prediction outcomes to varying degrees. Each cerebrovascular stenosis site in the ICA significantly affected the model's predictions, with the beginning of the ICA and the C1 segment having the highest weights. The MCA, ACA, and PCA also had a substantial influence, particularly the M1 segment of the MCA, A1 segment of the ACA, and P3 segment of the PCA. Conversely, the VA had minimal impact on the prediction results. For ischemic stroke patients, these anatomical sites are relatively common stenosis. Concurrently, the blood supply area of the middle cerebrovascular is also the responsible brain area for limb motor function, which is generally consistent with clinical practice. To some extent, it shows that acupuncture may achieve better therapeutic effect for patients involving these sites of cerebrovascular stenosis. Cerebral vascular stenosis, as the core cause of IS, not only serves as a criterion for assessing the severity of the patient's condition but also as a key factor affecting disease prognosis. Acupuncture is a promising complementary approach to conventional rehabilitation for the treatment of limb dysfunction after stroke. Recent studies have increasingly supported the role of acupuncture-induced vascular remodeling in alleviating limb motor dysfunction in patients with IS. Animal experiments have demonstrated that acupuncture effectively enhances cerebral hemodynamics by regulating blood flow velocity, vascular resistance index, and blood viscosity in patients with ischemic stroke [31, 32]. Additionally, acupuncture promotes angiogenesis through various signaling pathways, positively impacting cerebral perfusion, in addition to improving microcirculation by regulating key enzymes involved in cellular metabolism, thus improving the neurological prognosis of ischemic stroke [33]. The development of collateral circulation and the activity of brain microcirculation are both critical determinants of IS outcomes. Increasing evidence indicates that collateral circulation can predict the speed of infarction progression, the degree of reperfusion, the likelihood of hemorrhagic transformation, and various treatment opportunities. [34]. In the present study, we developed a predictive model based on the characteristics of cerebral vascular stenosis locations to identify regions more sensitive to acupuncture treatment. This work will provide a foundation for further exploration of the mechanism of cerebral vascular network remodeling in acupuncture treatment of IS.

**Fig. 7:**
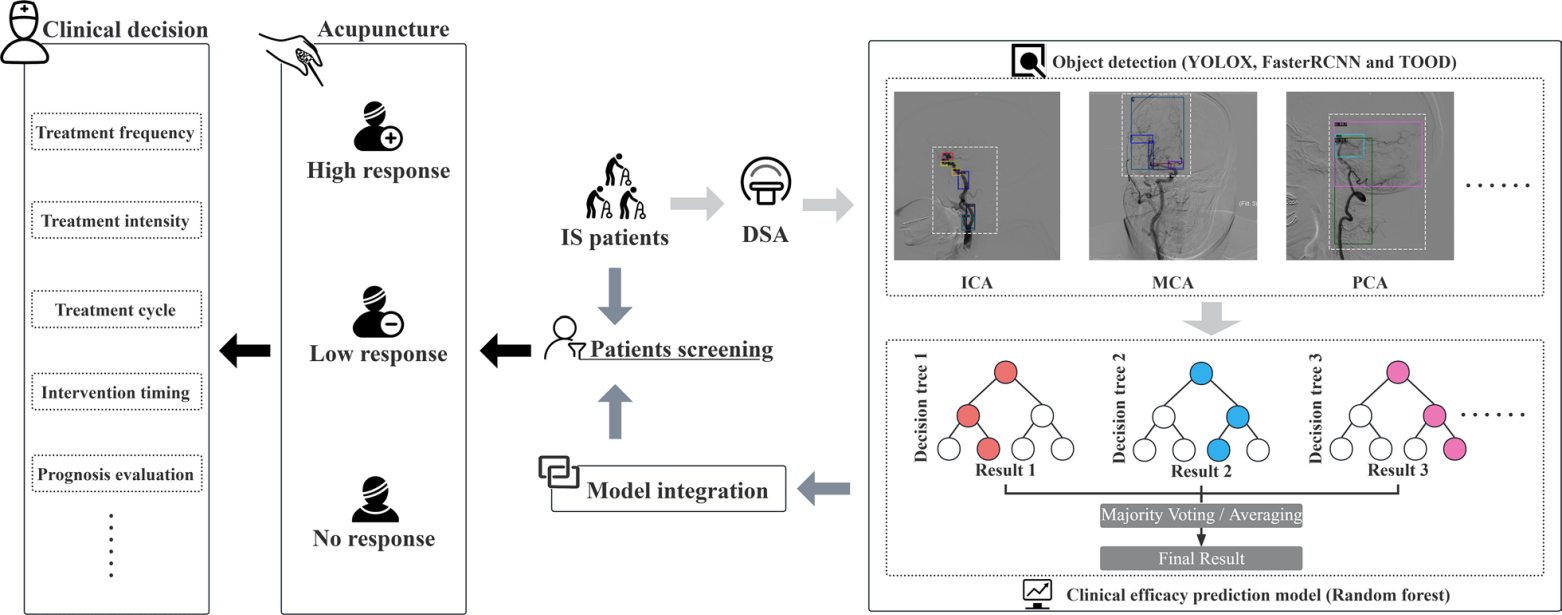
The clinical implementation path

We further conducted a stratified analysis of clinical data using object detection and efficacy prediction models. Initially, three object detection models were employed to identify cerebral blood vessel stenosis sites in DSA image data. Subsequently, the aggregated results from object detection served as feature inputs to predict the clinical efficacy. Given the numerous cerebral vascular classifications and limited sample size of the study, we used the RF algorithm to construct a clinical efficacy prediction model. This model demonstrated robust performance in classifying and predicting outcomes from high-dimensional, small-sample data [35, 36], as such, this study offers a prognostic model to predict the efficacy of acupuncture treatment for upper limb dysfunction in IS, based on DSA, the current gold standard for diagnosing cerebrovascular conditions. In clinical settings, this model could potentially equip clinicians with early stage insights into the benefits of acupuncture interventions, aiding in the formulation of treatment strategies. The clinical implementation pathway for this study is shown in Fig. 7.

In recent years, to advance precision treatment for stroke, an increasing number of researchers have employed machine learning to build clinical prediction models. Utilizing deep learning to develop prediction models based on image features has become a well-established analytical approach. For example, Kwon et al. predicted motor outcomes in IS patients using diffusion tensor imaging data [37]. Similarly, Nishi et al. employed deep learning–derived high-level neuroimaging features to predict clinical outcomes for large vessel occlusion [38]. These studies have established a potential link between neuroimaging features and stroke prognosis. However, no research has yet explored the relationship between cerebrovascular characteristics and stroke outcomes. This study utilized single-center retrospective clinical data to establish a connection between the efficacy of acupuncture in treating upper limb motor dysfunction following IS and cerebrovascular events, marking a novel endeavor. To enhance the data analysis, we integrated object detection models with a clinical efficacy prediction model to ensure the robustness of the final results. However, this study had certain limitations. First, while retrospective clinical data ensures consistency in acupuncture treatment regimens across patients, clinicians often tailor acupoint selection based on individual patient conditions. As such, future studies should incorporate datasets reflecting diverse acupuncture treatment regimens to enhance the applicability of this model in clinical settings. Second, due to the limited sample size, internal validation methods were employed to construct a clinical efficacy prediction model, potentially limiting its generalizability. Future research should involve the employment of multiple algorithmic models for stacking or comparison and expanding the sample size to enhance the generalization capacity of the predictive model.

## Conclusions

This study demonstrated the feasibility and reliability of using cerebrovascular characteristics derived from DSA to predict the efficacy of acupuncture in treating upper limb motor dysfunction following IS. Each site of cerebrovascular stenosis in the ICA significantly influenced the predictive outcomes of the model, particularly in the initial and C1 segments of the ICA, which emerged as pivotal predictors. These findings establish a quantifiable foundation for the application of acupuncture to treat upper limb motor dysfunction post-IS, thus aiding clinicians in tailoring personalized acupuncture protocols to enhance therapeutic outcomes and mitigate unnecessary medical resource expenditure. However, owing to the limitations of this study, studies with larger samples with higher-quality data are required to provide strong evidence to substantiate these findings comprehensively in future investigations.

## Data Availability

The datasets used and/or analysed during the current study are available from the corresponding author on reasonable request. The authors take full responsibility for the data, the analyses and interpretation, and the conduct of the research; that they have full access to all of the data; and that they have the right to publish any and all data, separate and apart from the guidance of any sponsor.
